# Membranes for Cation Transport Based on Dendronized Poly(Epichlorohydrin-Co-Ethylene Oxide). Part 2: Membrane Characterization and Transport Properties

**DOI:** 10.3390/polym13223915

**Published:** 2021-11-12

**Authors:** Alireza Zare, Xavier Montané, José Antonio Reina, Marta Giamberini

**Affiliations:** 1 Department of Chemical Engineering, Universitat Rovira i Virgili (URV), Av. Països Catalans, 26, 43007 Tarragona, Spain; alireza.zare@urv.cat; 2 Department of Analytical Chemistry and Organic Chemistry, Universitat Rovira i Virgili (URV), C/Marcel·lí Domingo s/n, 43007 Tarragona, Spain; joseantonio.reina@urv.cat

**Keywords:** Poly(epichlorohydrin-co-ethylene oxide), dendrons, cation-exchange membrane, wettability, methanol permeability, linear sweep voltammetry

## Abstract

In this paper, we report on the preparation and characterization of membranes out of two side-chain liquid crystalline copolymers, dendronized at two different extents (20 and 40%, CP20 and CP40, respectively). The membranes were characterized by atomic force microscopy (AFM), field-emission scanning electron microscopy (FESEM), contact angle (CA) analysis, and water uptake. Moreover, transport properties were studied by methanol and proton conductivity experiment and by linear sweep voltammetry (LSV). For the sake of comparison, the behavior of the grafted copolymers was compared with the unmodified copolyether CP0 and with Nafion 117. Results demonstrated that in CP20 and CP40, cation transport depends on the presence of defined cationic channels, not affected by water presence; the comparison between LSV experiments performed with different alkaline cations suggests that CP40 possesses channels with larger diameters and better-defined inner structures.

## 1. Introduction

During the last decades, the interest in finding more sustainable and respectful methods for generating energy such as artificial photosynthesis and electrochemical energy storage systems has greatly attracted the interest of the scientific community [1,2,3,4,5]. Proton-conducting membranes are one of the most important components in both energy systems taking into account their roles: separation of the two compartments and conduct selectively the protons from anode to cathode [6]. On the other hand, the design of proton-conducting membranes should also take into account how to avoid the permeation of active species. For example, in direct-methanol fuel cell and flow battery systems, the membrane must have a low permeability/crossover rate toward active methanol and metallic species [7,8].

Within the wide variety of materials that have been developed in the design of proton-conducting membranes, the synthesis of polymers capable for transport cations by mimicking the phenomena of ion transport that take place in biological membranes [9] was explored in recent decades.

In this direction, Percec et al. carried out an extensive investigation on the synthesis and subsequent self-assembly process of supramolecular dendrons, dendrimers, and dendronized polymers into columnar liquid-crystalline (LC) mesophases [10,11]. As a result, the intramolecular self-assembly process induces a helical conformation of the polymer backbones of these polymers, which are encircled by hydrophobic dendrons, providing to these materials the ability to transport cations in a selective manner, just as it happens in complex biological membranes [12,13].

The design of side-chain liquid-crystalline polyethers and polyamines that can work as ion transport channels due to the self-assembling of their polymeric columns, a process that is induced by the exo-recognition of the side-chain dendrons 3,4,5-tris[4-(n-dodecan-1-yloxy)bezyloxy]benzoate (Tap), is a topic which has been widely investigated by our research group [14,15,16,17]. The resulting self-organized structures show the complexity of its molecular design [16], which might be of value, for instance, for fuel cell applications. Additionally, membranes in which the polymer columns were homeotropically oriented have been prepared with the previously synthesized low molecular weight side-chain liquid-crystalline polymers (SCLCPs), which also showed proton transport results similar to those of Nafion® when they were tested as proton transport membranes [16,18,19,20]. However, in some cases the prepared membranes were brittle and exhibited poor mechanical properties, drawbacks that can be partially minimized by the preparation of hybrid membranes [18,19] or by using dendronized polymers with higher molecular weights such as Polyepichlorydrin or Poly(epichlorydrin-co-ethylene-oxide) (PECH-co-EO) [21]. Some of the most important factors that must be considered in the design of membranes that have the potential to transport cations are:

- The chemical composition of the polymer backbone, emphasizing which type of electron-withdrawing atom is present in polymer main chains.
- The amount of grafted mesogenic groups into the polymer backbone.
- The method (or combined methods) employed to favor the homeotropic alignment of the polymeric columns (thermal or light treatment, shearing, spinning) [22].

**Figure 1:**
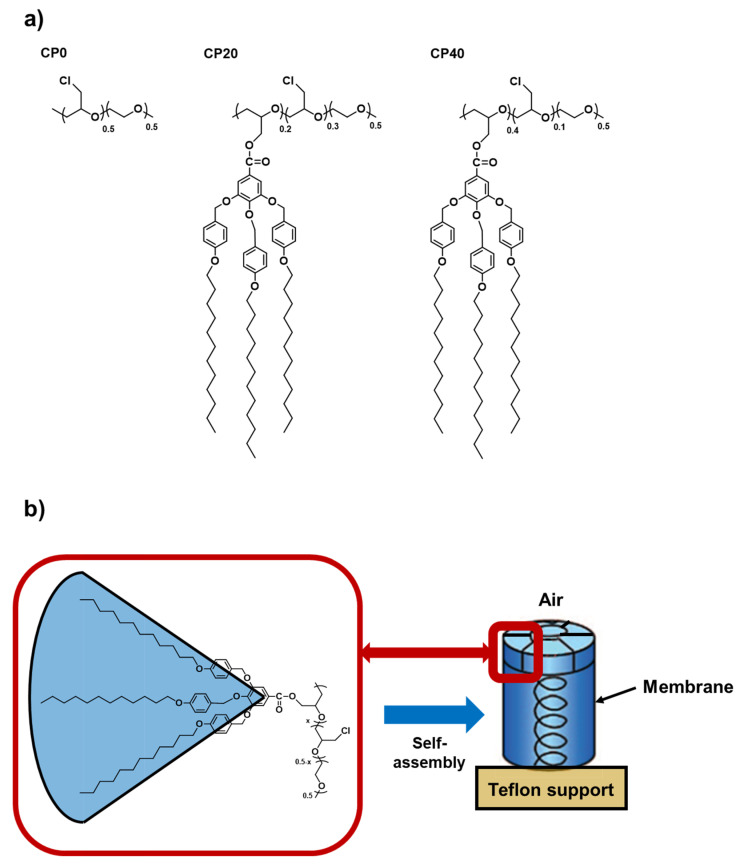
(**a**) Chemical structure of: CP0 (unmodified PECH-co-EO), CP20 (PECH-co-EO modified with 20% Tap dendron), and CP40 (PECH-co-EO modified with 40% Tap dendron). (**b**) Homeotropic alignment of the liquid-crystalline columns of the polymer to the membrane surface.

Among all of them, we have recently focused on studying the relationship between the orientation of distinct SCLCPs according to their degree of modification with Tap dendrons [23,24]. Furthermore, the analysis of how different parameters involved in the annealing process (annealing temperature, annealing time, and cooling rate) of the membranes prepared with dendronized Poly(epichloroydrin-co-ethylene oxide) that present 20% and 40% of grafted Tap dendrons have been deeply studied in Part 1 of this paper, entitled: “Membranes for cation transport based on dendronized Poly(epichlorohydrin-co-ethylene oxide). Part 1: The effect of the Dendron amount and column orientation on the copolymer mobility” [25]. In that study, PECH-co-EO units were chemically modified with Tap, and copolymers with different modification degrees of Tap were synthesized. The chemical structure of PECH-co-EO (CP0) and the copolymers modified with 20% (CP20) and 40% (CP40) of Tap, which are used for the preparation of membranes, are depicted in Figure 1a. Moreover, the resulting structure of the homeotropically oriented PECH-co-EO columns modified with Tap are shown in Figure 1b.

As reported, the decrease in the cooling rate results in an improvement in the orientation of the polymeric columns perpendicular to the membrane surface. Nonetheless, the most relevant factor is the selection of the annealing temperature, which should be as close as possible to the clearing temperature (T_c_) determined by differential scanning calorimetry (DSC) since a higher mobility of the copolymer structure is favored when the temperature is increased. Moreover, this study showed that the orientation method produces an increment of the crystalline fractions and the melting temperature (T_m_) of these SCLCPs. Even though the analysis of all these properties is crucial, the assessment of the membranes in transport systems will allow for evaluating their efficacy in applications related to proton transport. Thus, the preparation and characterization of membranes derived from two side-chain liquid crystalline copolyethers that differ in the amount of Tap side-chain dendrons (20 and 40%, respectively) have been carried out in present work. According to Part 1 of this paper, membranes were exposed to a thermal treatment that induces the self-assemble of the polymer chains into columnar structures. In this sense, the main goal of this study is to investigate how the presence of two different amounts of grafted dendrons can affect the transport capacity of oriented membranes by means of methanol and proton permeability, together with linear sweep voltammetry (LSV) analysis. The results obtained were compared with the commercial membrane Nafion 117. Moreover, other properties such as the wettability, the morphology, and the topography of unoriented and oriented membranes have been examined; in this way, one can understand the evolution of the self-assembly process that occurs in these ionic channels and the final organization of the polymeric columns, for the purpose of optimizing the design of membranes, and obtain good performance in operative conditions.

## 2. Materials and Methods

### 2.1. Materials

Inorganic and organic compounds were purchased from Sigma Aldrich (Sigma Aldrich Química, Madrid, Spain) and Fisher Scientifics (Fisher Scientific Spain, Madrid, Spain) and used as received. All the solvents were purchased from Scharlab (Scharlab, S.L., Barcelona, Spain). Potassium 3,4,5-tris[4-(n-dodecan-1-yloxy)bezyloxy]benzoate (Tap) was synthesized according to the previous report [16]. Poly(epichlorohydrin-co-ethylene oxide) (P(ECH-co-EO) 1:1, M_w_ = 5.01 × 10^5,^ M_n_= 1.08 × 10^5^ determined by gel permeation chromatography) was purchased from Sigma-Aldrich and used as received. The Nafion^®^ 117 membrane from DuPont was provided by Fuel Cell Etc (College Station, TX, USA) and cleaned before using [26]. For the cleaning treatment of Nafion, the following steps were performed: The membrane was washed in a hydrogen peroxide aqueous solution 5% for 1h at 80 °C to remove residual impurities. Then, the membrane was washed in 0.5 M sulfuric acid solution for 1 h at 80 °C. The membrane was placed in boiling deionized water for 1 h to remove the remaining impurities. Finally, the membrane was immersed in a container of H_2_O_2_ 3% for 1.5 h at 80 °C, deionized water for 1 h at 80 °C, H_2_SO_4_ 0.5 M for 1h at 80 °C, and washed in deionized water for 1 h at 80 °C, respectively. Before experiments, the membrane was kept in deionized water at room temperature.

The chemical modification of P(ECH-co-EO) was performed as described in Part 1 [25]. In the present paper, we focused our attention on the polymer modified at 20% and 40%, which are labelled as CP20 and CP40. On the other hand, the unmodified PECH-co-EO was labelled as CP0.

### 2.2. Membrane Preparation and Characterizations

Membranes were prepared by immersion precipitation method. The modified copolymer was dissolved in THF (30% *w*/*w*). After that, the homogeneous solution was casted by a casting machine (K-paint applicator, RK Paintcoat Instruments Ltd., Litlington, UK) on a FEP (Fluorinated Ethylene Propylene) sheet support with a controlled thickness (gap size 300 μm). Then, the support including the wet film on top was immersed in a bath of Milli-Q water in which the polymeric membrane was formed with an asymmetric structure. After 24 h, the formed membrane was dried overnight at room temperature. Additionally, the membranes were vacuum dried at room temperature 48 h before weighing. 

The thickness of membranes was measured using a micrometer with a sensitivity of 2 μm. The measurements were carried out at various points, and the membranes were found to have constant thickness.

To achieve homeotropically oriented structure of modified copolymer, the polymeric membrane (approx. 2 cm diameter) was placed on a hot stage (Linkam TP92, Linkam Scientific Instruments Ltd., Tadworth, UK) for the *baking* process, described as follows. For annealing, membranes were heated up to 140 °C. They were kept at the same temperature for 30 min. Then, they were slowly cooled (0.1 °C/min) to 107 °C where they were kept for 120 h. Finally, the membranes were allowed to cool to room temperature at 10 °C/min. Finally, the uniform membranes were left at room temperature for 1h and then detached from FEP support. For scale-up, the same procedure was performed in a Hewlett Packard 5890 Series II Gas Chromatograph oven (Hewlett Packard, Palo Alto, Santa Clara, CA, USA).

The static contact angles of water drops on both membrane surfaces were measured by Dataphysics OCA 15EC (DataPhysics, Fildertstadt, Germany). The contact angle was measured immediately after placing the water drop (3 µL) on the membrane surface and the contact angle was calculated from a digital image by SCA software included in the apparatus; for each test reported, at least three drops of water were used.

Water uptake of membranes was calculated according to the Equation (1) through immersing dry membranes into deionized water at room temperature for 72 h. Membranes were weighed before and after immersing in water.

(1) $$\mathrm{Water}\;\mathrm{uptake}\;\left(\%\right)=\frac{W_{\mathrm{wet}}-{\;W}_{\mathrm{dry}}}{W_{\mathrm{dry}}}\times 100$$

W_wet_ and W_dry_ refer to the weights of the wet and dry membranes, respectively.

The AFM images were recorded with an Agilent 5500 Environmental Atomic Force Microscope (Agilent Technology, Santa Clara, CA, USA) equipped with an extender electronics module, which enables phase imaging in a Tapping Mode. All images were recorded in a tapping mode using 75 Al g Budget sensor (freq. 71 kHz) silicon cantilever (thickness = 3 μm) with a force constant at 3 N/m and a resonance frequency of 71 kHz The scan rate was typically 0.7–2 Hz. All images (1 μm × 1 μm) were measured at room temperature, in unfiltered air. The microscope was placed on an active vibration isolation chamber (Agilent Technology), which was further placed on a large sturdy table to eliminate external vibration noise. The Nanotec WSxM 5.0 Develop 9.1 Image Browser Scanning Probe Microscopy23 (Julio Gómez Herrero and José María Gómez Rodríguez) was used for the roughness analysis of the images [27]. RMS roughness and kurtosis were calculated out of the AFM images of five different regions for each sample.

Field-emission scanning electron microscopy (FESEM) was performed by means of Scios2 microscope (Thermo Fisher Scientific, Waltham, MA, USA). Electrons were accelerated at 5.00 kV, the working distance comprised between 3 and 7 mm and ETD or T2- high resolution secondary electrons detectors were used. Membranes were previously criofractured in liquid nitrogen in order to observe their cross sections.

The methanol permeability was measured using a permeability cell, which included two compartments (feed and stripping, respectively) that were connected through the membrane. The effective membrane area was 0.86 cm^2^. The feed and the stripping volumes were 200 mL, which were filled up with 2 M methanol solution (feed compartment) and milli-Q water (stripping compartment), respectively. After 24, 48, 96, and 168 h, the methanol content was determined by means of a Hewlett Packard 6890 Series Gas Chromatograph (Hewlett Packard, Palo Alto, USA), J&W DB-624 GC Column, 25 m, 0.20 mm, 1.12 µm, 7-inch cage (Agilent Technology, Santa Clara, USA) and He as flowing gas. Results were interpolated from a standard curve obtained from previous experiments: Am/Aiso = 0.3458 Cm/Ciso + 0.033 (R^2^ = 0.9992), where Am is the area corresponding to the methanol peak and Aiso is the area of the isopropanol peak which was used as an internal standard in 0.05 M concentration; Cm and Ciso are the methanol and the constant isopropanol molar concentrations, respectively. The calibration line was obtained by using 22 points with a methanol concentration ranging between 0.01 and 0.7 M. All samples were analysed in triplicate. The detection limit was calculated according to [28] and was found as 0.0879, which corresponds to a methanol concentration equal to 4.4 × 10^−3^ M.

Proton permeability was performed using a Teflon set-up that comprised two compartments, the feed and stripping solutions, separated by the tested membrane. Samples for these measurements were placed between two compartments giving a total membrane area equal to 0.66 cm^2^. The solution volume in each compartment was 200 mL. The measurements were carried out in the ambient temperature (approx. 25 °C). The pH of the stripping solution was measured every 10 s by an Orion 4 Star PH/ISE Multimeter (Thermo Fisher Scientific, Waltham, MA, USA). For the proton transport experiments, the initial feed solution was 0.1 M HCl aqueous solution and the stripping solution 0.1 M aqueous solution of the corresponding salt: LiCl, NaCl or KCl. The calculations of the methanol and proton permeability were carried out in accordance with the following equations.

The permeability coefficient, *p* (cm s^−1^), can be described by the equation [29]:

(2) $$-\ln\frac{C_{f}}{C_{0}}=\frac{Ap}{V_{f}}t$$

where *C*_0_ (mol l^−1^) is the initial concentration of the feed solution and *C_f_* (mol L^−1^) is the feed concentration calculated from the stripping solution at time *t* (s):

(3) $$C_{f}=C_{0}-C_{s}$$

*V_f_* is the feed volume (mL) and *A* is the actual membrane area (cm^2^).

Under steady-state conditions, proton flux was calculated by Fick’s First Law:

(4) $$J=\frac{P\Delta C}{l}$$

where *l* (cm) is the membrane thickness and Δ*C* is the difference in concentration between the initial feed solution and the final stripping solution. In our experimental conditions, *C*_0_ was much greater than the final stripping concentration, so we considered $\Delta C\approx C_{0}$.

The permeability *P* (cm^2^ s^−1^) is defined as:

(5) P=pl

The flux is then related to the permeability coefficient as:

(6) $$J=pC_{0}$$

In the case of protons, data were fitted according to Equation (1) in the time range 15–120 h.

Linear sweep voltammetry was performed according to [19] using Autolab PGstat204 in potentiostatic mode with current ranging from 100 mA to 100 μA, potential range from 0 V to 5 V, step 0.01 V and scan rate 0.0013 V s^−1^. The experimental set-up for linear sweep voltammetry measurements is shown in Figure S1. The distance between the membrane and reference electrodes (Ag/AgCl) was 1.13 cm. The sample was placed in a Teflon frame with a hole giving a total membrane area equal 0.5 cm^2^. The measurements were performed at the ambient temperature. The solution volume in each compartment was 200 mL. One series of measurements was applied to one membrane including HCl, LiCl, NaCl, and KCl, respectively. An experiment for each specific cation includes five consecutive current–voltage measurements at different time frames: 5, 10, 20, 40, and 60 min.

## 3. Results

In this study, in order to elucidate how the Tap amount affects the final material characteristics, we focused our attention on the copolymers modified at 20% (CP20) and 40% (CP40), respectively. Their characteristics were compared with the unmodified copolymer CP0. Nafion 117 was also used as a reference for proton transport properties.

### 3.1. Morphological Characterization, Wettability and Water Uptake

In Part 1 of this paper [25], we showed that grafting of Poly(epichlorohydrin-co-ethylene oxide) with Tap group, induced crystallization of the copolymer main chain, and that this effect was more evident in the copolymer-based oriented membranes. Crystallinity calculated from DSC data lay around 30% in the unoriented samples, whereas it increased to 38% in oriented CP20 and to 52% in oriented CP40. It has to be remembered that the orientation corresponds to the homeotropic alignment of the liquid crystalline columns to the membrane surface. Therefore, the microstructure of these materials can be expected quite complex, since crystalline, liquid crystalline, and amorphous regions coexist.

**Figure 2:**
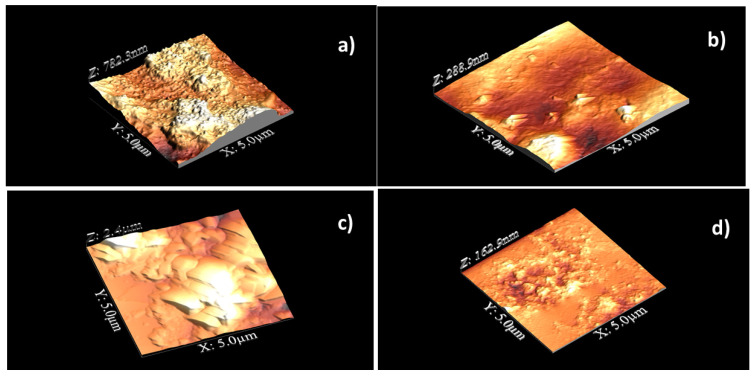
Topographic 3-D images of CP20 unoriented (**a**) and oriented (**b**), of CP40 unoriented (**c**) and oriented (**d**). Scanned area: 5 µm × 5 µm.

Figure 2 shows the AFM topographic images of CP20 unoriented (a) and oriented (b), of CP40 unoriented (c) and oriented (d).

From the 3-dimensional topographic images, we can see that the surface of unoriented CP20 (Figure 2a) exhibits protruding bundles, with apparent diameter around 100 nm, surrounded by small flat areas; after orientation (Figure 2b), extreme flattening of the surface is observed. As for CP40, on the surface of the unoriented sample (Figure 2c), one can see more extended flat areas; after orientation (Figure 2d), the material clearly re-organizes, and small grains appear. A small granular structure was found within and outside the dendritic regions of semi-crystalline PEO and amorphous polymethylmethacrylate (PMMA) phase-separated blends [30].

Table 1 shows RMS roughness and kurtosis for CP20 and CP40, as calculated from five AFM images for each sample.

As shown in Table 1, in the case of CP20, orientation determines a considerable decrease in RMS roughness, which is the standard deviation of height; the opposite is found for CP40, though the average RMS value of its oriented sample remains lower than oriented CP20. Kurtosis, which illustrates the sharpness of the surface height distribution, increases for both samples upon orientation, i.e., peaks predominate over the valleys: this effect is more pronounced for CP40, where we pass from a mesokurtic surface (unoriented sample), to a leptokurtic one (oriented sample). Kurtosis values larger than 3.0 generally indicate a surface with almost quantized height values.

Membrane wettability was determined by water contact angle (CA) measurements. The experiments were performed on unoriented samples, as well as on membranes oriented by thermal treatment, as previously described. In addition, CA was determined both on the side in direct contact with the FEP support and on the air side. Results are depicted in Table 2.

Unmodified CP0 showed hydrophilic behavior, as expected based on its chemical structure, which contains polar ether linkages. Unoriented CP20 exhibited higher CA values on both sides, though they were still lower than 90°, i.e., the presence of the dendrons somewhat decreased the hydrophilic character of the copolymer; differently, unoriented CP40, with higher amount of Tap group, exhibited hydrophobic behavior on both sides. Upon orientation, both dendronized copolymers showed water CA higher than 90°, i.e., clearly had hydrophobic surfaces, and no substantial difference was found between the air and the Teflon side. One has to consider that two effects contribute to wettability: The former is the chemical composition of the material, which can be more or less affined to water; the latter is the surface morphology, i.e., its roughness. Given the same chemical composition, the effect of a roughened surface is to magnify the wetting properties of the membrane. Therefore, if one considers a water-repelling material, the rougher the surface, it will be more strongly so [31]. In our previous studies on dendronized copolymer-based membranes, we found that thermal orientation induced an increase in water CA, and we attributed this evidence to the dendron exposure on the membrane surface in the oriented material, since it constitutes the hydrophobic portion of the copolymer. This was considered as an indirect evidence of the fact that the dendron anchoring to the air surface drives the orientation [21]. In the case of CP40, apart from this effect, one should also take into account that there is a strong increase in roughness in the oriented sample, which, together with the higher number of dendrons possibly exposed to the surface, contributes to greatly enhance hydrophobicity.

The wettability change of CP20 and CP40 on orientation also reflects on the water uptake (WU) after 72 h. The results of these measurements are shown in Table 3.

As expected, the WU of CP0 after 72 h has a considerable value (Table 3). Chemical modification with the Tap group remarkably decreases the WU of both CP20 and CP40, probably due to two factors, i.e., the presence of the hydrophobic, aromatic, and aliphatic groups in the grafted moiety, as well as the induced crystallinity in the main chain. Essentially, it has been demonstrated for many polymers that crystallinity hinders moisture sorption [32,33]. After orientation, water uptake is further reduced to very low values, which is in line with the wettability decrease (Table 2) and the increase in crystallinity. As for methanol permeability, in the case of CP0, methanol could be detected in the stripping compartment during the permeability experiment, and gave a permeability equal to 1.79 × 10^−6^ cm^2^ s^−1^. Nevertheless, in the case of oriented CP20 and CP40, even after 168 h the methanol concentration in the stripping compartment was below the detection limit [28], i.e., it was lower than 4.4 × 10^−3^ M. Therefore, the characteristics of dendronized CP20 and CP40 could also offer advantages as far as methanol crossover in direct-methanol fuel cells (DMFCs) is concerned.

Water contact angle was also determined on oriented CP20 and CP40 membranes after water uptake and proton permeability tests, which results are reported in Table S1. In general, no significant changes were observed (Table S1), except for CP40, which water contact angle was slightly reduced after the proton permeability test; however, the remaining characteristic was of a clearly hydrophobic surface.

We also attempted the characterization of CP0, CP20, and CP40 by FESEM. Unfortunately, in the case of CP0 and CP20, strong damage was provoked to the samples by the radiation under the applied conditions. Essentially, the acquisition of information on soft matter insulators, such as polymers, can be a difficult task, due to sample charging and radiation damage. This drawback has been traditionally overcome by coating the polymer with a thin conductive layer, but this strategy masks tiny surface details [34]. Therefore, in the case of CP0, CP20 unoriented and CP20 oriented, we could not reach magnification higher than 800 in the experimental conditions used. Figure S2 shows the CP0 (a), CP20 (b), and CP20-oriented (c) cross-section images. Operating in these conditions allowed us to obtain a global image of the cross-section of CP0 and CP20. In the case of CP0, the cross-section appeared quite smooth and somewhat folded (Figure S2a); however, a much rougher and heterogenous cross-section could be distinguished in unoriented CP20 (Figure S2b). Orientation of CP20 seemed to produce a more homogeneous cross-section (Figure S2c).

On the other hand, intriguing results were obtained when FESEM analysis was performed on unoriented and oriented CP40 samples. In this case, the working distance could be lowered to about 3 mm and we could obtain magnification as high as 350 k, thus revealing a higher resistance of these samples to radiation damage.

**Figure 3:**
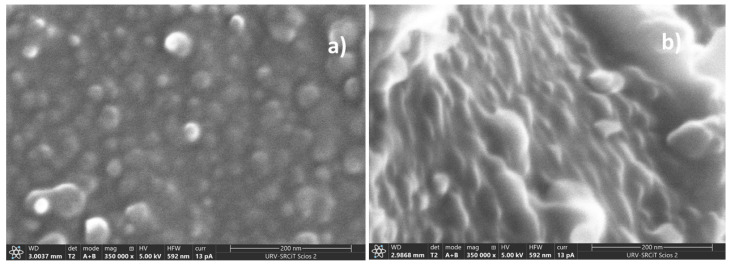
FESEM images of membrane cross-sections of: CP40 (**a**) and CP40-oriented (**b**).

Figure 3 depicts FESEM images of CP40 (a) and CP40-oriented (b) membrane cross-sections.

**Figure 4:**
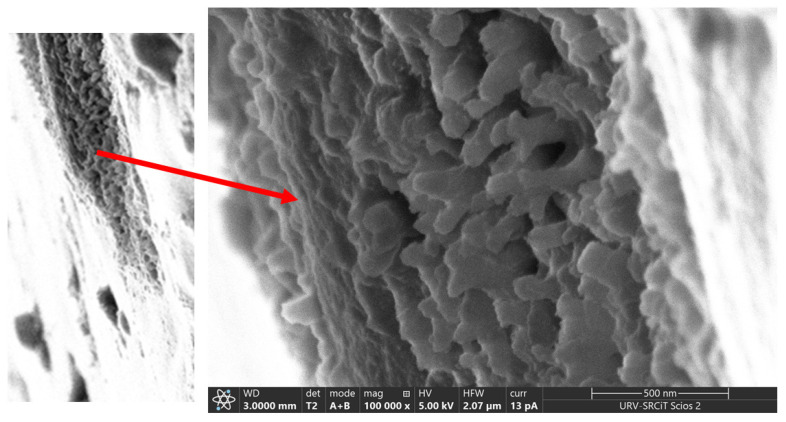
FESEM image of the scratched surface of CP40 oriented membrane. Left: membrane surface with scratched portion; Right: zoom of the scratched area.

The cross section of CP40 exhibits spherical particles, with diameters ranging between 20 and 50 nm (Figure 4). The particles look more elongated in the case of the oriented sample, resembling cylindrical aggregates with diameters comprised approximately between 7 and 40 nm. Particularly interesting is the image of the scratched surface of CP40 oriented membrane (Figure 4), where bundles with diameters ranging around 50 nm can be clearly seen.

These bundles can be related to the “ionic cables” formed by aggregates of oriented columns, and responsible for the ionic transport, which existence was suggested also by TEM observations performed on dendronized poly(epichlorohydrin) [21].

### 3.2. Transport Properties

In general, ionic transport in membranes occurs through the system of channels and pores, which size depends on the nature of the polymer and hydration degree. The transport of ions in the narrow channels is responsable for limiting ionic conductivity: Therefore, narrow channels are usually called the “bottle neck” [35]. In a previous paper, we investigated the proton and sodium transport mechanism of oriented membranes based on P(ECH-co-EO), modified at 36% with the same dendron Tap, by in situ Raman spectroscopy coupled with amperometric experiment [36]. We found that the polyether backbone is mainly involved in both cation transport, in agreement with our previous hypotheses. Moreover, in the case of protons, conduction also occurs through an additional coordination site which lies on the lateral ester group that actively interacts with protons during their transport. The investigated membranes exhibited water uptake as low as 4 ± 1%; in addition, in Raman spectra, the range characteristic of OH stretching of water showed no significant changes. Therefore, we concluded that cation transport in these membranes is not significantly influenced by the presence of water.

Table 4 shows the proton permeability of CP0, CP20, and CP40 in the presence of different cations in the stripping compartment. For sake of comparison, proton permeability was also determined for Nafion 117 in the presence of NaCl in the stripping compartment.

In our previous papers, we already made the hypothesis of a cation antiport mechanism [29] in the case of dendronized columnar copolymers, which seemed to be confirmed by the agreement between the proton concentration increase in the stripping solution and the simultaneous sodium concentration increase in the feed solution, when the permeability experiment was performed with this species in the stripping compartment [37]. The coupled Raman-LSV studies performed on CP modified at 36% [36] also gave an inside into the possibility of conducting cations via antiport mechanism. It was observed that only protons can be transported via both the main chain and the lateral ester groups, whereas sodium ions only by interaction with the main chain. Having those findings in mind, the antiport transport in similar system could take place even within a single channel where the proton would by-pass the bigger cation transported in opposite direction, through an ester lateral group. Additionally, proton transport in proton permeability experiments should be generally limited by the stripping cation permeability which, in the case of CP20 and CP40, is expected to depend on the size of the polyether ion channel. In addition, we have to take into account that CP20 and CP40 are partially crystalline [25], which also contributes to limit cation permeability. Differently, in the case of CP0 and Nafion 117, transport is expected to occur through the channels formed by hydration of the respective membranes: Actually, they exhibited comparable proton permeabilities, around 10^−6^ cm^2^ s^−1^, in the experiment where the stripping phase contained Na^+^. In the case of this experiment, considerably lower values were found for proton permeability of CP20 and CP40, which were comparable to the ones found for poly(epichlorohydrine) 72% modified with the same dendron [21]. 

No proton permeability at all could be detected for CP20 and CP40 when the stripping compartment contained KCl, suggesting that this cation is too big for the transport to occur through the oriented channels. On the other hand, in all cases, lower proton permeability values were calculated when Li^+^ was contained in the stripping phase, despite its lower crystallographic radius, which might seem anomalous. Essentially, one should remember that Li^+^ exhibit peculiar features with respect to other alkaline cations: Li^+^ possesses a very high hydration number, which determines the highest hydrated radius of the considered series and the weakest electrostatic interactions [38]. In addition, it was found that the diffusion coefficient of Li^+^ in cation-exchange membranes strongly depends on the water content [39]. Therefore, limited Li^+^ transport through CP20 and CP40 membranes could be due to a combination of the membrane low affinity to water as well as to the big size of the hydrated cation. Moreover, it has been generally found that in cation-exchange membranes, the cation diffusion coefficients and ionic conductivity increase in the sequence Li^+^ < Na^+^ [40]; in general, the cation diffusion coefficients increase the alkaline metal atomic mass, due to higher water mobility and lower effective radius of the hydrated cation.

When membranes containing hydrated non-specific channels are considered, the charge balance is assured by the parallel diffusion of cations and anions, though anions are characterized by smaller mobility. Nevertheless, it has been found that, in the case of electrolytes transferred to an acid filled compartment in a permeability experiment, the charge balance is reached mainly by a mutual diffusion of protons and alkali metal ions, though a parallel diffusion of cations and anions exist [41]. Given the higher diffusion of the cations, the contribution of anion diffusion can be neglected; therefore, proton permeability depends on the co-ion mobility. Additionally, we need to take into consideration the water swelling of the membrane and the hydrophilic nature of the used materials. These two factors can significantly affect the ionic transport giving a misguided impression of an actual ionic transport. As a result of the membrane swelling, undefined channels filled with absorbed water are formed, similarly to those in Nafion. In this scenario, cation flow is restricted in very small range only by the quantity of the absorbed water and the channel structure. This could justify the trend of proton permeabilities found for CP0, where no specific columnar ionic channels exist. 

**Figure 5:**
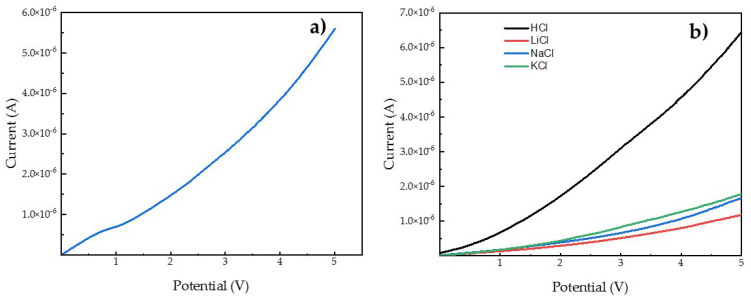
Linear sweep voltammetry curve of CP40 and 0.1M NaCl as electrolyte (**a**) and example of curves for different monovalent cations for CP20 (**b**).

In order to characterize the ionic transport in terms of selectivity, linear sweep voltammetry (LSV) measurements were performed. Our previous studies [19,20,36] confirmed that the linear sweep voltammetry is a suitable tool to study transport through the non-ionomeric membranes. Differently from the permeability test, the ionic transport measured in a setup for LSV allows cation transport in one direction. For the experiment, 0.1 M solutions of hydrochloric acid and different monovalent chlorides were used to establish the selectivity of used membranes. For most cases of CP-based membranes, the three-region division of the LSV curve was not evident and the I-V curves have a linear tendency. Only in the case of CP40 and 0.1M NaCl solution the ohmic, limiting current, and overlimiting regions were observed (see Figure 5). This fact confirms the permselective nature of CP20 and CP40, what was expected based on results obtained for CP copolymer modified in 36% [36]. Permselectivity of membranes indicates that ionic transport occurs only via cation flux.

Table 5 summarizes the data obtained from the LSV experiments. As can be seen from the resistance value per membrane’s area, an increasing order was observed, giving a CP0 < CP40 < CP20 order for the different membranes. It aligns very well with the tendency observed in permeability test. This fact confirms that the one direction conductivity of cations depends on the effective cavity of the channel, also for the LSV experiments. In order to describe a selectivity of used membranes, a selectivity factor was calculated as the ratio between conductance (inverse resistance) for specific alkali cation and conductance of proton × 100. Therefore, the higher the selectivity factor, the lower the selectivity of the membrane toward the proton, with respect to the considered cation. It was noticed that the order for the selectivity factor was as follows: Na^+^ > K^+^ > Li^+^ for CP0 and CP20 and K^+^ > Na^+^ > Li^+^ for CP40. The information coming from the selectivity order of cationic transport gives an idea of the channel size. It seems that the cavity of ionic channels for CP20 is more likely to fit the sodium cation than other tested cations, whereas in the case of CP40, the conduction of potassium was greater than for other alkali cations; this suggests that the greater modification degree of the copolymer causes the formation of channels with larger diameter. When compared with Nafion 117 membrane and to CP0, where the ionic transport is affected by the presence of water and the channel structure is not well defined, the selectivity between different cations depends on ionic forces in the case of ionomeric Nafion 117, or by hydration radius, as described in the section regarding the permeability test.

## 4. Conclusions

Membranes derived from two side-chain liquid crystalline copolyethers, differing in the amount of Tap side-chain dendrons (20 and 40%, respectively) have been prepared and characterized in terms of morphology, wettability, water uptake, methanol permeability, and transport properties. Proper orientation of these membranes was achieved by means of a thermal treatment, and the morphological characterization was carried out on both the unoriented and oriented samples as well as on the unmodified copolyether. In general, AFM showed that orientation leads to a decrease in roughness for CP20 and an increase for CP40, the latter being much less rough in any case; in both samples, hydrophobicity increased with membrane orientation. Water uptake was considerably lower than found for the unmodified copolyether, being furtherly reduced on orientation; in both grafted copolymers, no methanol permeability was detected at all. FESEM analysis showed bundles with diameters ranging around 50 nm in the cross-section of the oriented CP40 sample; unfortunately, experiments with similar magnification could not be performed on CP20 nor CP0, because of sample charging and radiation damage.

The permeability test for the oriented CP20 and CP40 demonstrated an evident tendency related to the presence of defined cationic channels not affected by water presence. The higher modified polymer CP40 showed better permeability results, probably as an effect of the better inner structure of channels across the entire structure, due to a greater number of Tap groups supporting the column. Linear sweep voltammetric studies confirmed the permselective nature of the new membranes, meaning the ionic transport occurs only via cation transport across the membrane. The best cationic performance was observed in the case of proton and then for sodium/potassium and lithium cations.

## Figures and Tables

**Table 1:** RMS roughness and kurtosis from AFM images for CP20 and CP40 samples.

Sample	RMS Roughness (µm)	Kurtosis
CP20	128 ± 26	3.6 ± 1.3
CP20 oriented	44 ± 11	4.6 ± 1.6
CP40	0.44 ± 0.08	2.7 ± 0.1
CP40 oriented	16 ± 3	4.8 ± 1.1

**Table 2:** Water contact angle values of unmodified Poly(epichlorohydrin-co-ethylene oxide) (CP0), CP20, and CP40 membranes.

Sample	Contact Angle (°)	
	Unoriented ^a,b^	Oriented ^a,b^
CP0	72 ± 1	-
CP20	87 ± 2 ^a^, 89 ± 1 ^b^	104.5 ± 0.9 ^a^, 106.5 ± 0.4 ^b^
CP40	96.3 ± 0.3 ^a^, 97.0 ± 0.5 ^b^	131.0 ± 0.8 ^a^, 129.8 ± 0.3 ^b^

^a^ Air side; ^b^ Teflon side.

**Table 3:** Water uptake (%) after 72 h, and methanol permeability after 168 h, of unmodified Poly(epichlorohydrin-co-ethylene oxide) (CP0), CP20, and CP40 membranes.

Sample	**WU (%), Unoriented**	WU (%), Oriented	Methanol Permeability (cm^2^ s^−1^)
CP0	28	-	1.79 × 10^−6^
CP20	10 ± 0.5	2.5 ± 0.5	Not Detected
CP40	7.5 ± 0.5	2.0 ± 0.5	Not Detected

**Table 4:** Proton permeabilities of CP0, CP20, and CP40 in the presence of different cations in the stripping compartment.

Sample	Proton Permeability (cm^2^ s^−1^)		
	Cation in Stripping Phase		
	Li^+^	Na^+^	K^+^
CP0	(2.7 ± 0.2) × 10^−6^	(3.4 ± 1.6) × 10^−6^	(2.2 ± 0.1) × 10^−5^
CP20	(1.2 ± 0.3) × 10^−8^	(3.1 ± 0.5) × 10^−8^	Not detected
CP40	(1.4 ± 0.2) × 10^−7^	(3.4 ± 0.5) × 10^−7^	Not detected
Nafion 117	-	(7.7 ± 0.2) × 10^−6^	-

**Table 5:** A summary of data obtained from LSV experiments.

Sample Name	Thickness (µm)	Electrolyte	Resistance (kΩ/cm^2^)	Selectivity (%)
Nafion	200	HCl	0.03	-
		LiCl	0.16	16.5
		NaCl	0.11	24.2
		KCl	0.08	31.0
CP0	360	HCl	863.9	-
		LiCl	1470.59	58,7
		NaCl	1108.65	77,8
		KCl	1175.78	73,5
CP20	150	HCl	3333.33	-
		LiCl	16260.16	20.5
		NaCl	12269.94	27.2
		KCl	12578.62	26.5
CP40	200	HCl	1492.54	-
		LiCl	8230.45	19.4
		NaCl	2597,40	51.3
		KCl	2564.10	57.4

## Data Availability

The data presented in this study are available on request from the corresponding author. The data are not publicly available due to their deposition at an offline disk, MEMTEC group, URV, Tarragona, Spain.

## Supplementary Materials

The following are available online at https://www.mdpi.com/article/10.3390/polym13223915/s1, Figure S1: The experimental set-up for lineal sweep voltammetry measurements. Anode (+) is working electrode (WE), Cathode (−) is counter electrode (CE), S is sensitive electrode, and RE is reference electrode; Figure S2: FESEM images of the membrane cross-sections: CP0 (a); CP20 (b); CP20-oriented (c); Table S1: Water Contact angle as determined on oriented CP20 and CP40 membranes after Proton Permeability experiment (PP) and Water Uptake test (WU).

## References

[1] Berardi S., Drouet S., Francàs L., Gimbert-Suriñach C., Guttentag M., Richmond C., Stoll T., Llobet A. (2014). Molecular artificial photosynthesis. Chem. Soc. Rev..

[2] Keijer T., Bouwens T., Hessels J., Reek J.N.H. (2021). Supramolecular strategies in artificial photosynthesis. Chem. Sci..

[3] Cheng Y.Y., Fückel B., MacQueen R.W., Khoury T., Clady R.G.C.R., Schulze T.F., Ekins-Daukes N.J., Crossley M.J., Stannowski B., Lips K. (2012). Improving the light-harvesting of amorphous silicon solar cells with photochemical upconversion. Energy Environ. Sci..

[4] Sazali N., Salleh W.N.W., Jamaludin A.S., Razali M.N.M. (2020). New Perspectives on Fuel Cell Technology: A Brief Review. Membranes.

[5] Zhang H., Sun C. (2021). Cost-effective iron-based aqueous redox flow batteries for large-scale energy storage application. J. Power Sources.

[6] Shin D.W., Guiver M.D., Lee Y.M. (2017). Hydrocarbon-Based Polymer Electrolyte Membranes: Importance of Morphology on Ion Transport and Membrane Stability. Chem. Rev..

[7] Sun C., Negro E., Nale A., Pagot G., Vezzù K., Zawodzinski T.A., Meda L., Gambaro C., Di Noto V. (2021). An efficient barrier toward vanadium crossover in redox flow batteries: The bilayer [Nafion/(WO3)x] hybrid inorganic-organic membrane. Electrochim. Acta.

[8] Parthiban V., Akula S., Sahu A.K. (2017). Surfactant templated nanoporous carbon-Nafion hybrid membranes for direct methanol fuel cells with reduced methanol crossover. J. Membr. Sci..

[9] Shen Y.X., Saboe P.O., Sines I.T., Erbakan M., Kumar M. (2014). Biomimetic membranes: A review. J. Membr. Sci..

[10] Percec V., Heck J. (1991). Liquid crystalline polymers containing mesogenic units based on half-disc and rod-like moieties—4. Side chain liquid crystalline polymethylsiloxanes containing hemiphasmidic mesogens based on 4-[3,4,5-tri-(alkan-1-yloxy)benzoate]biphenyl groups. J. Polym. Sci. Part A Polym. Chem..

[11] Sun H.-J., Zhang S., Percec V. (2015). From structure to function via complex supramolecular dendrimer systems. Chem. Soc. Rev..

[12] Rosen B.M., Wilson C.J., Wilson D.A., Peterca M., Imam M.R., Percec V. (2009). Dendron-mediated self-assembly, disassembly, and self-organization of complex systems. Chem. Rev..

[13] Sherman S.E., Xiao Q., Percec V. (2017). Mimicking complex biological membranes and their programmable glycan ligands with dendrimersomes and glycodendrimersomes. Chem. Rev..

[14] Bhosale S.V., Rasool M.A., Reina J.A., Giamberini M. (2013). New Liquid crystallne Columnar Poly(epichlorohydrin-co-ethylene oxide) Derivatives Leading to Biomimetic Ion Channels. Polym. Eng. Sci..

[15] Montané X., Bhosale S.V., Reina J.A., Giamberini M. (2015). Columnar Liquid Crystalline Polyglycidol De-rivatives: A Novel Alternative for Proton-Conducting Membranes. Polymer.

[16] Montané X., Bogdanowicz K.A., Colace G., Reina J.A., Cerruti P., Lederer A., Giamberini M. (2016). Advances in the Design of Self-Supported Ion-Conducting Membranes-New Family of Columnar Liquid Crystalline Polyamines. Part 1: Copolymer Synthesis and Membrane Preparation. Polymer.

[17] Šakalytė A., Reina J.A., Giamberini M. (2013). Liquid Crystalline Polyamines Containing Side Dendrons: To-ward the Building of Ion Channels Based on Polyamines. Polymer.

[18] Bogdanowicz K.A., Rapsilber G.A., Reina J.A., Giamberini M. (2016). Liquid Crystalline Polymeric Wires for Selective Proton Transport, Part 1: Wires Preparation. Polymer.

[19] Bogdanowicz K.A., Sistat P., Reina J.A., Giamberini M. (2016). Liquid Crystalline Polymeric Wires for Selective Proton Transport, Part 2: Ion Transport in Solid-State. Polymer.

[20] Montané X., Bogdanowicz K.A., Prats-Reig J., Colace G., Reina J.A., Giamberini M. (2016). Advances in the Design of Self-Supported Ion-Conducting Membranes—New Family of Columnar Liquid Crystalline Pol-yamines. Part 2: Ion Transport Characterisation and Comparison to Hybrid Membranes. Polymer.

[21] Bogdanowicz K.A., Bhosale S.V., Li Y., Vankelecom I.F.J., Garcia-Valls R., Reina J.A., Giamberini M. (2016). Mimicking Nature: Biomimetic Ionic Channels. J. Membr. Sci..

[22] Lee W.J., Kwac L.K., Kim H.G., Chang J.-H. (2021). Thermotropic liquid crystalline copolyester fibers according to various heat treatment conditions. Sci. Rep..

[23] Teruel-Juanes R., Bogdanowicz K.A., Badia J.D., de Juano-Arbona V.S., Graf R., Reina J.A., Giamberini M., Ribes-Greus A. (2021). Molecular Mobility in Oriented and Unoriented Membranes Based on Poly[2-(Aziridin-1-yl)ethanol]. Polymers.

[24] Teruel-Juanes R., Pascual-Jose B., Graf R., Reina J.A., Giamberini M., Ribes-Greus A. (2021). Effect of Dendritic Side Groups on the Mobility of Modified Poly(epichlorohydrin) Copolymers. Polymers.

[25] Zare A., Pascual-Jose B., De la Flor S., Ribes-Greus A., Montané X., Reina J.A., Giamberini M. (2021). Membranes for cation transport based on dendronized Poly(epichlorohydrin-co-ethylene oxide). Part 1: The effect of the Dendron amount and column orientation on the copolymer mobility. Polymers.

[26] Prater K.B. (1994). Polymer electrolyte fuel cells: A review of recent developments. J. Power Sources.

[27] Horcas I., Fernandez R., Gomez-Rodriguez J.M., Colchero J., Gomez-Herrero J., Baro A.M. (2007). WSXM: A software for scanning probe microscopy and a tool for nanotechnology. Rev. Sci. Instrum..

[28] Garcia-Valls R. (1995). Nous Materials en Tècniques de Separació D’elements Lantànids: Membranes Polimètriques Activades i Materials Inorgànics per a Cromatografia. Ph.D. Thesis.

[29] Mulder M. (2003). Basic Principles of Membrane Technology.

[30] Ferreiro V., Douglas J.F., Amis E.J., Karim A. (2001). Phase Ordering in Blend Films of Semi-crystalline and Amorphous Polymers. Macromol. Symp..

[31] Wenzel R.N. (1936). Resistance of solid surfaces to wetting by water. Ind. Eng. Chem..

[32] Scheirs J., Long T.E. (2003). Modern Polyesters: Chemistry and Technology of Polyesters and Copolyesters.

[33] Bascheka G., Hartwiga G., Zahradnikb F. (1999). Effect of water absorption in polymers at low and high temperatures. Polymer.

[34] Wandrol P., Slouf M. (2017). Polymer imaging in SEM—Charge, damage and coating free. Microsc. Microanal..

[35] Yaroslavtsev A.B., Stenina I.A., Golubenko D.V. (2020). Membrane materials for energy production and storage. Pure Appl. Chem..

[36] Bogdanowicz K.A., Pirone D., Prats-Reig J., Ambrogi V., Reina J.A., Giamberini M. (2018). In Situ Raman Spectroscopy as a Tool for Structural Insight into Cation Non-Ionomeric Polymer Interactions during Ion Transport. Polymers.

[37] Tylkowski B., Castelao N., Giamberini M., Garcia-Valls R., Reina J.A., Gumí T. (2012). The importance of orientation in proton transport of a polymer film based on an oriented self-organized columnar liquid-crystalline polyether. Mater. Sci. Eng. C.

[38] Cotton F.A., Wilkinson G. (1980). Advanced Inorganic Chemistry.

[39] Garrido L., Pozuelo J., López-González M., Yan G., Fang J., Riande E. (2012). Influence of the Water Content on the Diffusion Coefficients of Li+ and Water across Naphthalenic Based Copolyimide Cation-Exchange Membranes. J. Phys. Chem. B.

[40] Volkov V.I., Chernyak A.V., Golubenko D.V., Tverskoy V.A., Lochin G.A., Odjigaeva E.S., Yaroslavtsev A.B. (2020). Hydration and Diffusion of H, Li, Na, Cs Ions in Cation-Exchange Membranes Based on Polyethylene- and Sulfonated-Grafted Polystyrene Studied by NMR Technique and Ionic Conductivity Measurements. Membranes.

[41] Stenina I.A., Sistat P., Rebrov A.I., Pourcelly G., Yaroslavtsev A.B. (2004). Ion mobility in Nafion-117 membranes. Desalination.

