# Supplementary material for: Membranes for Cation Transport Based on Dendronized Poly(Epichlorohydrin-Co-Ethylene Oxide). Part 2: Membrane Characterization and Transport Properties
Source: Polymers (Basel). 2021 Nov 12;13(22):3915. doi: 10.3390/polym13223915 (PMC8619552; doi:10.3390/polym13223915)
Supplement: Supplementary file 1 [file polymers-13-03915-s001.zip › polymers-1417811-supplementary.pdf]

Supplementary Material

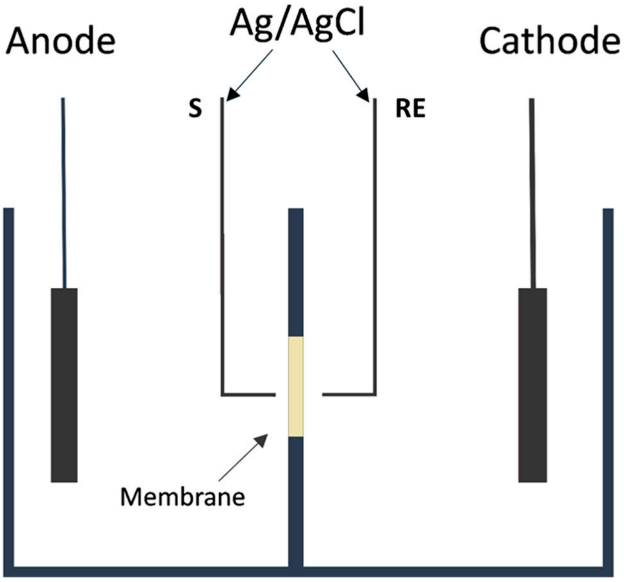

Figure S1. The experimental set-up for linear sweep voltammetry measurements. Anode (+) is working electrode (WE), Cathode (-) is counter electrode (CE), S is sensitive electrode and RE is reference electrode.

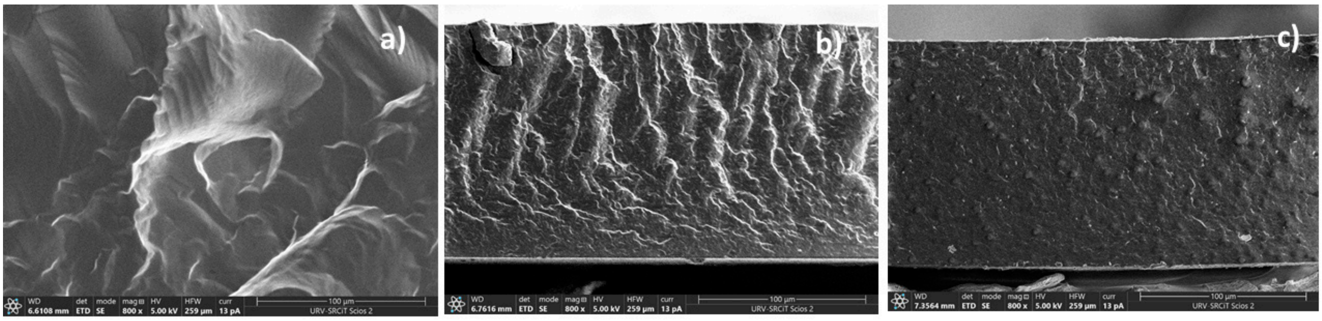

Figure S2. FESEM images of the membrane cross-sections: CP0 (a); CP20 (b); CP20 oriented (c).

Table S1 Water Contact angle as determined on oriented CP20 and CP40 membranes after Proton Permeability experiment (PP) and Water Uptake test (WU).

Sample	Modification (%)	Contact angle (°)	
		Oriented membrane after PP	Oriented membrane after WU
**CP20**	20%	106 ± 2 [a], 107 ± 1 [b]	106 ±2 [a], 108 ±3 [b]
**CP40**	40%	124 ± 2 [a], 122 ± 2 [b]	130 ± 3 [a], 130 ± 2 [b]

[a] Air side; [b] Teflon side
